# N-acetylaspartate (NAA) induces neuronal differentiation of SH-SY5Y neuroblastoma cell line and sensitizes it to chemotherapeutic agents

**DOI:** 10.18632/oncotarget.8454

**Published:** 2016-03-29

**Authors:** Carmela Mazzoccoli, Vitalba Ruggieri, Tiziana Tataranni, Francesca Agriesti, Ilaria Laurenzana, Angelo Fratello, Nazzareno Capitanio, Claudia Piccoli

**Affiliations:** ^1^ Laboratory of Pre-Clinical and Translational Research, IRCCS-CROB, Referral Cancer Center of Basilicata, Rionero in Vulture, Pz, Italy; ^2^ Department of Clinical and Experimental Medicine, University of Foggia, Foggia, Italy

**Keywords:** N-acetylaspartate, neuroblastoma, cell differentiation, chemotherapy

## Abstract

Neuroblastoma is the most commonly extra-cranial solid tumor of childhood frequently diagnosed. The nervous system-specific metabolite N-acetylaspartate (NAA) is synthesized from aspartate and acetyl-CoA in neurons, it is among the most abundant metabolites present in the central nervous system (CNS) and appears to be involved in many CNS disorders. The functional significance of the high NAA concentration in the brain remains uncertain, but it confers to NAA a unique clinical significance exploited in magnetic resonance spectroscopy. In the current study, we show that treatment of SH-SY5Y neuroblastoma-derived cell line with sub-cytotoxic physiological concentrations of NAA inhibits cell growth. This effect is partly due to enhanced apoptosis, shown by decrease of the anti-apoptotic factors survivin and Bcl-xL, and partly to arrest of the cell-cycle progression, linked to enhanced expression of the cyclin-inhibitors p53, p21^Cip1/Waf1^ and p27^Kip1^. Moreover, NAA-treated SH-SY5Y cells exhibited morphological changes accompanied with increase of the neurogenic markers TH and MAP2 and down-regulation of the pluripotency markers OCT4 and CXCR4/CD184. Finally, NAA-pre-treated SH-SY5Y cells resulted more sensitive to the cytotoxic effect of the chemotherapeutic drugs Cisplatin and 5-fluorouracil.

To our knowledge, this is the first study demonstrating the neuronal differentiating effects of NAA in neuroblastoma cells. NAA may be a potential preconditioning or adjuvant compound in chemotherapeutic treatment.

## INTRODUCTION

Neuroblastoma (NB) is the most common extracranial solid tumor and the most frequently diagnosed neoplasm during infancy [1]. NB is characterized as a biologically and clinically heterogeneous tumor that ranges from spontaneously regressing growth to aggressively malignant and incurable. The tumor originates from embryonic neural crest cells that are committed to development of the sympathetic nervous system. Although major advances have been made in surgery and chemotherapy of NB, the morbidity and mortality remain high. Thus far, the molecular mechanisms responsible for the pathogenesis of NB remain elusive [2].

N-acetyl-L-aspartic acid (NAA) is the N-acetylated derivative of the amino acid L-aspartate (ASP), present in the central nervous system (CNS) of humans and other animals and its concentration is between 8 mM and 12 mM in the human brain [3–5]. Although NAA functions in the central nervous system are still a subject of study, it has been demonstrated that it is the first precursor for the biosynthesis of the neurotransmitter N-acetylaspartylglutamate (NAAG) [6–8]. NAA is synthesized via enzymatic acetyl-CoA-mediated acetylation of aspartate by L-aspartate-N-acetyltransferase 8 like (NAT8L) into the mitochondria of neurons and transferred to the cytosol [9–10]. NAA is metabolized to acetate and aspartate in the CNS by N-acetyl-L-aspartate aminohydrolase (also known as aspartoacylase) which is expressed in glial cells and oligodentrocytes but not in neurons or astrocytes [10–11]. This restricted distribution of the biosynthetic and metabolic enzymes accounts for the high concentrations of NAA within the neurons of the CNS and low concentrations outside of the neurons. The enzymatic deacetylation of NAA is the primary source of free acetate for the biosynthesis of fatty acids which are used in the myelination of neurons of the mammalian CNS during postnatal development [12–16].

Due to its high concentration, the regional levels of NAA can be monitored non-invasively by proton magnetic resonance spectroscopy (MRS) [17]. NAA is considered a marker of neuronal integrity and viability and the knowledge of its levels in the CNS cell helps to interpret the changes in magnetic resonance spectra observed in a variety of CNS disorders [18–19]. Most of these studies have shown a decrease in NAA concentrations in the affected brain areas, with the notable exception of Canavan disease which involves accumulation of NAA throughout the brain [20]. In neurodegenerative diseases reduced NAA concentration may be associated with irreversible loss of neurons, reversible neuronal or mitochondrial dysfunction [21–26].

In the current study, we used the SH-SY5Y human catecholaminergic neuroblastoma cell line, a third-generation subclone of SK-N-SH [27], a well-established model system to study the initial phases of neuronal differentiation. We show for the first time that the NAA-treatment induces SHSY5Y cells differentiation similar to that induced by Retinoic Acid (ATRA) treatment even though to lesser extent. Consequently, NAA treated cells appear more sensitive to conventional drugs. This observation could open up new avenues in treating neurological cancers.

## RESULTS

### Effect of NAA on cell viability

**Figure 1:**
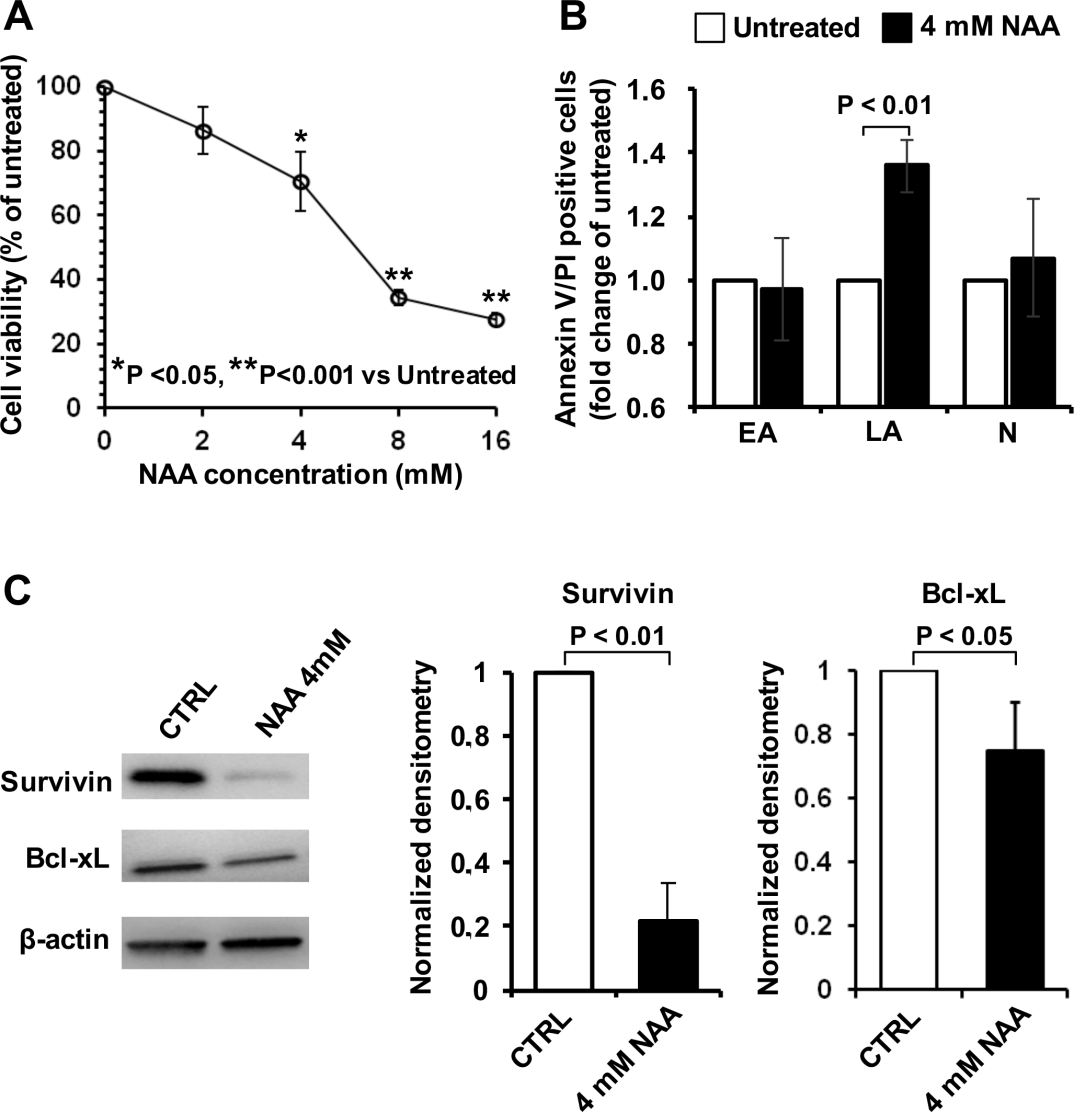
Effect of NAA on cell viability and apoptosis in SH-SY5Y cells (**A**) Dose-dependence effect of NAA treatment on cell viability. Cells were exposed for 72 h to the indicated concentrations of NAA and viability determined by MTS assay. Cell viability is expressed as the percentage (%) of untreated cells. The data shown are means (± SEM) of 6 independent experiments. (**B**) Measurement of early apoptotic (EA), late apoptotic (LA) and necrotic (N) cells performed by flow-cytometry after staining cells with Annexin-V and Propidium Iodide. Cells were incubated with 4 mM NAA for 72 h. Data, expressed as percentage of total events analysed, are the means (± SEM) of three independent experiments. (**C**) Protein expression levels of the anti-apoptotic factors, Bcl-xL and Survivin, assayed by Western blotting in untreated and 4 mM NAA-treated cells for 72 h (left panel); β-actin served as loading control. Graph bars on the right show the average (± SEM) of data resulting from densitometric analysis of three independent blots.

In order to evaluate the effect of NAA on cell viability, SH-SY5Y cells were treated with different concentrations of NAA and analyzed by MTS assay. Figure 1A shows that 72 h of NAA treatment caused a dose-dependent decrease of cell viability becoming significant at 4 mM where around 30% of cells displayed reduced viability as compared to untreated cells. On this basis, we chose 4 mM NAA onward as optimal concentration able to induce significant modifications in cell viability but, at the same time, preserving it enough so as to consent further analysis. In agreement with the results of the MTS assay, 4 mM NAA-treatment for 72 h resulted in SH-SY5Y cells in a significant 30% increase of annexin-V/PI positive cells indicating an NAA-mediated stimulation of late apoptosis (Figure 1B). No significant differences in the percentage of early apoptotic or necrotic cells were observed following NAA treatment. To confirm the induction of apoptosis in NAA-treated SH-SY5Y cells, the expression of the anti-apoptotic factors Survivin and Bcl-xl was evaluated by immune-blotting. As shown in Figure 1C, both Survivin and Bcl-xl were significantly reduced (by 80% and 30% respectively) in NAA-treated cells as compared to the untreated control.

### Nuclear magnetic resonance spectroscopy of NAA-treated cultured cells

**Figure 2:**
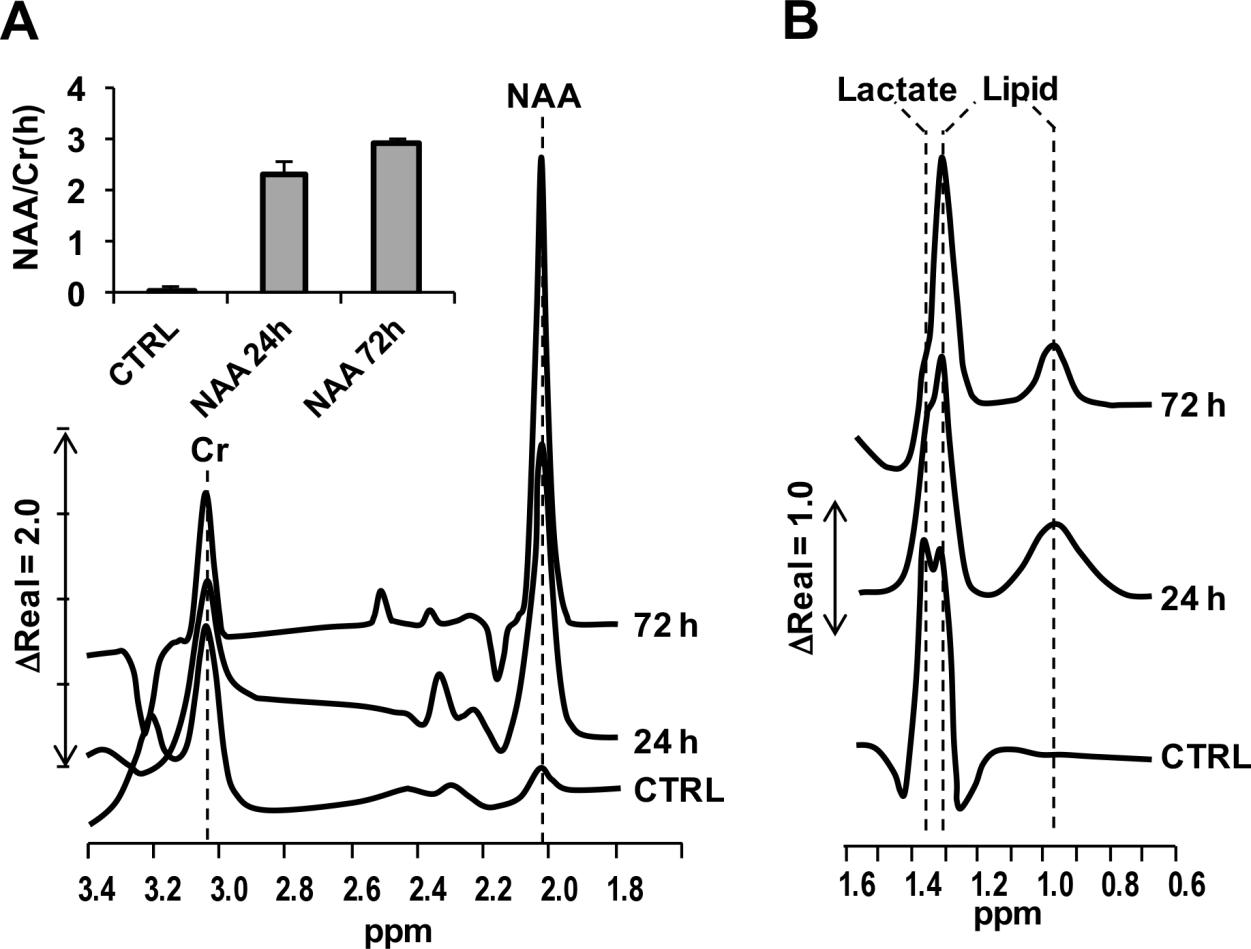
NMR spectrograms of NAA-treated SH-SY5Y cells Cultured SH-SY5Y cells were untreated (CTRL) or incubated 4 mM NAA for 24 h and 72 h; at the end of the incubation-time cells were extensively washed with NAA-less fresh medium and subjected to 3T-NMR specroscopic scanning as described in Materials and Methods. (**A**) Spectrogram in the 1.8–3.4 ppm range showing the peaks of NAA and creatine (Cr); the inset quantifies the peak-height of NAA normalized to that of Cr. (**B**) Spectrogram in the 0.6–1.6 ppm range showing the peaks of lactate and lipids [33].

The uptake of NAA in SH-SY5Y cells was ascertained exploiting the well-defined NMR spectroscopic feature of the methyl group of the acetyl moiety of NAA [17]. Figure 2 shows the NMR spectrograms attained on cultured SH-SY5Y cells following 24 h and 72 h treatment with 4 mM NAA. The spectra were recorded after extensive removal of the NAA-containing media and the peak height of creatine (at around 3.0 ppm) taken as normalizing metabolite. As compared with untreated cells, where the endogenous content of NAA (peaking at about 2.0 ppm) was negligible, compound-treated cells displayed a large and progressive increase of the relative content of NAA (Figure 2A) (see also [28]). Importantly, peaks in the 1.4–0.9 ppm, diagnostic for lactate and lipids [29], unveiled a progressive decline of the lactate-related peak (i.e. decreased glycolysis) and increase of the lipid-related peak at about 0.95 ppm (i.e. enhanced lipogenesis) (Figure 2B). These results show that the administered NAA is taken up by SH-SY5Y, likely via the Na^+^-dependent high-affinity dicarboxylate transporter NaDC3 as described elsewhere [30], and metabolized.

### Effect of NAA on cell growth

**Figure 3:**
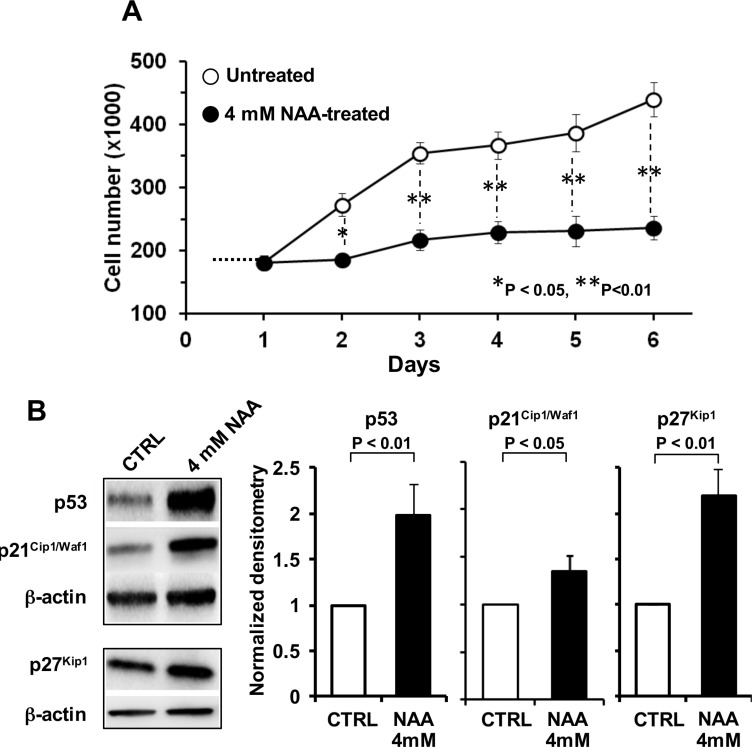
Effect of NAA on cell proliferation and cell cycle (**A**) Growth curve of SH-SY5Y cells in the presence or absence of NAA. Cells were grown in the standard culture medium and treated with 4 mM of NAA. After treatment with NAA, cells were trypsinized, harvested and number of viable cells was determined in triplicate. (**B**) Protein expression levels of p53, p21 and p27, assayed by Western blotting in untreated and 4mM NAA-treated cells for 72 h (left panel); β-actin served as loading control. Graph bars on the right show the average (± SEM) of data resulting from densitometric analysis of three independent blots.

Consistent with the observed effect on cell viability, 4 mM NAA caused also a marked impairment in the growth rate of SH-SY5Y when the compound-treatment was prolonged for 6 days (Figure 3A). The observed decline of the growth rate was supported by the observation that the cell-cycle blocker p53, p21^Cip1/Waf1^ and p27^Kip1^ resulted significantly up-regulated in NAA-treated cells (Figure 3B).

### Effect of NAA on the cell differentiation state

**Figure 4:**
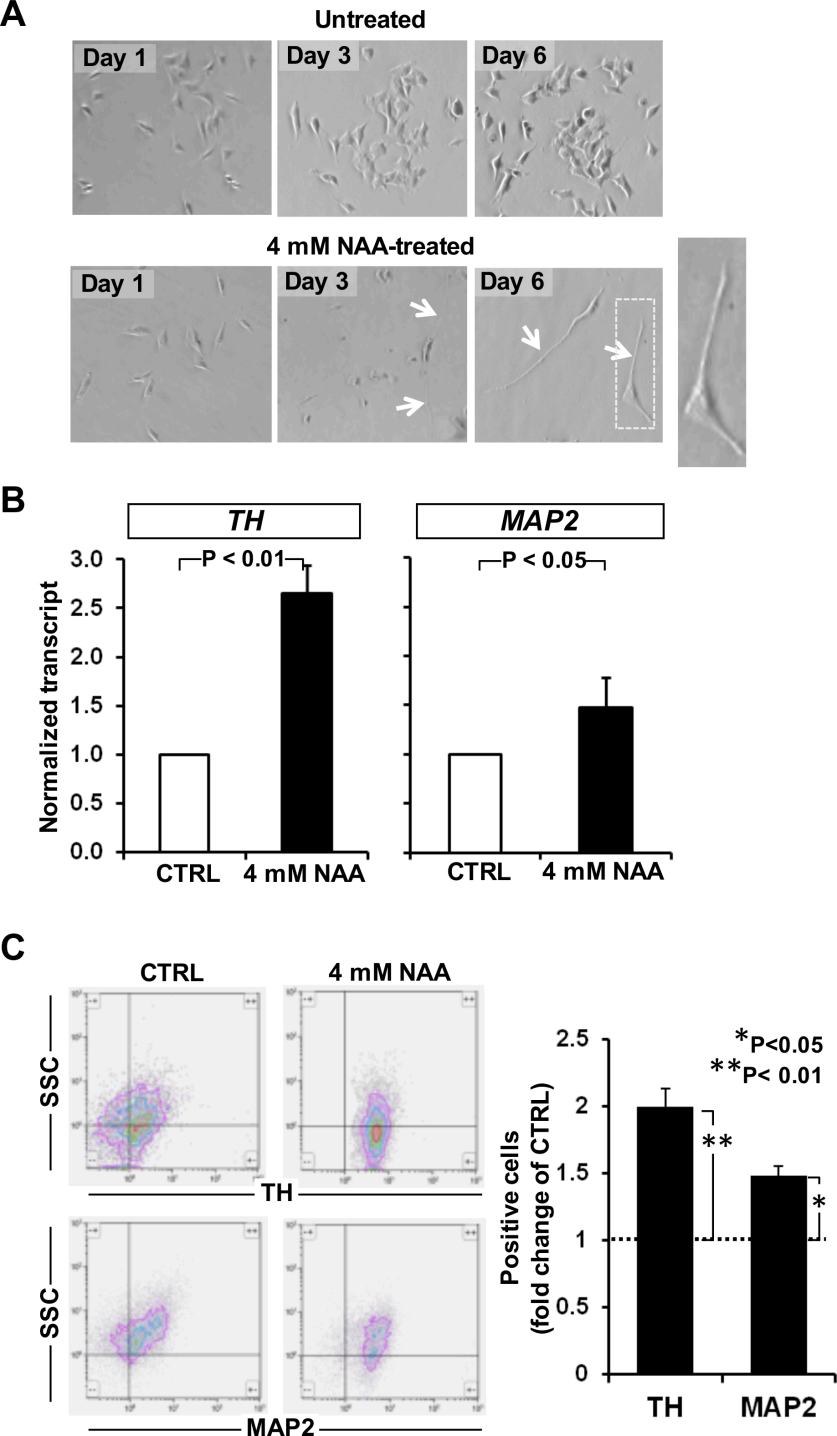
Effect of NAA on cell morphology and neuronal marker expression (**A**) Morphological comparison between undifferentiated and differentiated SH-SY5Y cells after 3 and 6 days of 4 mM NAA treatment. NAA treatment - induced dramatic morphological changes can be seen including smaller cell bodies and substantial increases in neurite outgrowth and complexity. (**B**) SH- SY5Y cells were treated with 4 mM NAA for 72 h. mRNA was harvested and expression of neuronal differentiation genes (TH, MAP2) has been analysed. (**C**) Flow cytometric detection of TH and MAP2 in neural SH-SY5Y cell line. Representative experiment out of three performed is shown. The differentiation markers TH and MAP2 were significantly upregulated after 3 days of treatment. Graph bars on the right show the average (± SEM) of data resulting from quantification of TH and MAP2 signals from three independent experiments.

Interestingly, observation of the SH-SY5Y over the 6 days of NAA-treatment revealed progressive morphological changes hallmarked by elongated shape with protrusion of two-three cellular processes thus acquiring a neurite-like morpho-phenotype. These changes resembled those caused by 10 μM ATRA, a well-known neurogenic differentiation inducer of SH-SY5Y [31] (Figure 4A). The state of differentiation irrespective of the compound used was highly dependent on cell density, with more pronounced differentiation at lower cell densities (not shown).

To confirm the acquisition of a neuron phenotype in NAA-treated SH-SY5Y cells of a neuron phenotype two marker genes of differentiation into neuronal-like cells, the tyrosine hydroxylase (TH) and the microtubule-associated protein 2 (*MAP2*) were evaluated. As shown in Figure 4B the transcript levels of both *TH* and *MAP2*, assessed by q-RT-PCR, were significantly increased by 2.5 and 1.5 fold respectively as compared with untreated cells. The enhanced expression of the two neurogenic markers was further verified at the protein level by immune-florescence flow-cytometry resulting in good agreement with what observed at the transcriptional level (Figure 4C).

**Figure 5:**
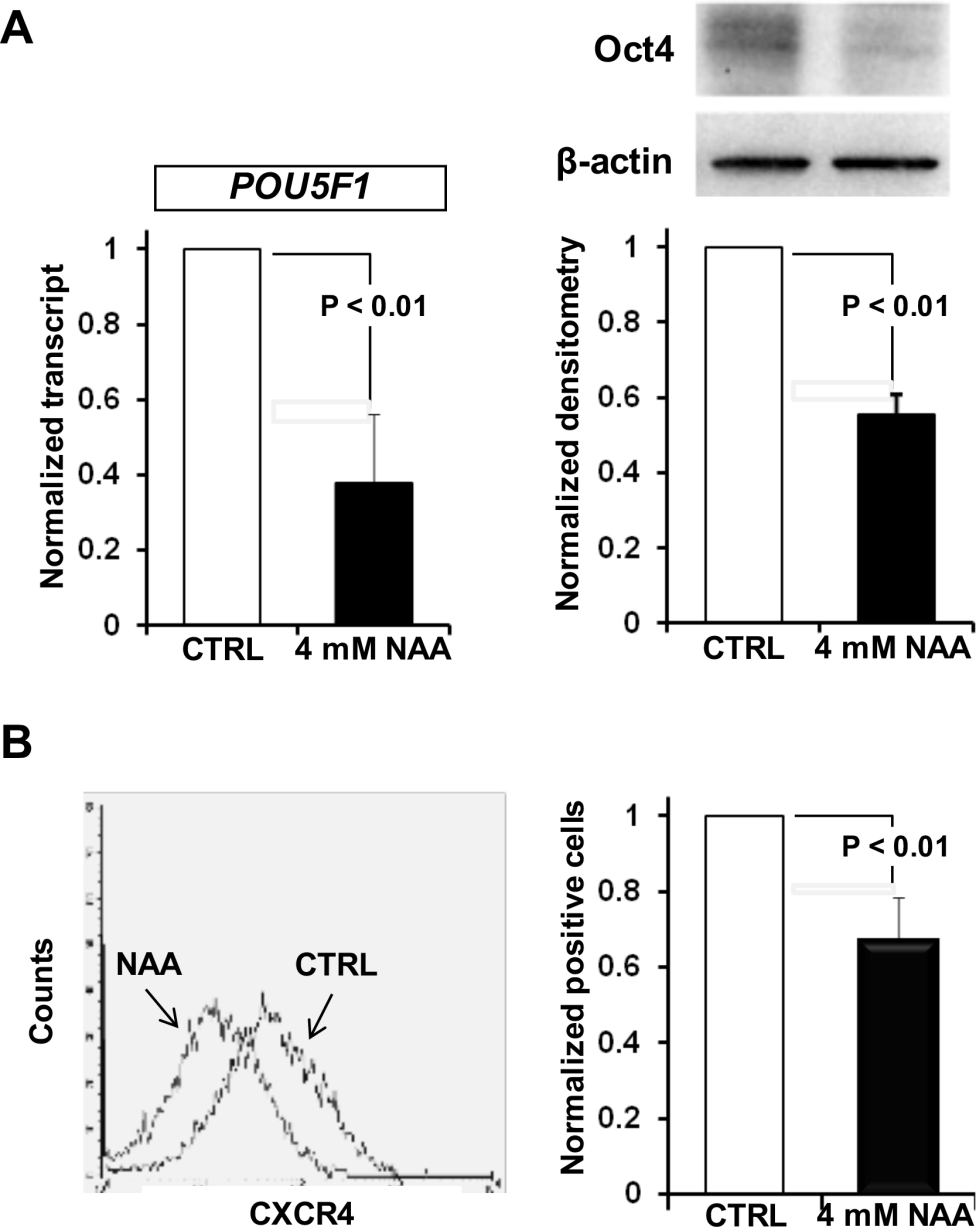
Effect of NAA on stemness marker expression (**A**) Oct4 is down-regulated after NAA treatment. Relative levels of Oct4 mRNA in SH-SY5Y treated cells are shown as histograms. Oct4 mRNA expression was quantified by RT-PCR compared with GAPDH. Western blot analysis of Oct4 protein levels after NAA treatament. β-actin served as loading control. Graph bars on the right show the average (± SEM) of data resulting from densitometric analysis of three independent blots. (**B**) Flow cytometric analysis of surface marker CXCR4(CD184) after 4 mM NAA treatment. One representative experiment out of three performed is shown. Graph bars on the right show the average (± SEM) of data resulting from quantitation of CD184 signal from three independent experiments.

To further support the loss of the undifferentiated state in NAA-treated SH-SY5Y the expression levels of the “stemness” marker OCT4 and of the C-X-C chemokine receptor 4 (CXCR4/CD184), a marker of undifferentiated state, were evaluated. As shown in Figure 5A, 5B, both the expression levels of OCT4 (at transcriptional and translational level) and CXCR4/CD184 were significantly reduced following 72 h treatment of SH-SY5Y cells with 4 mM NAA.

### Effect of co-treatment of NAA and antineoplastic drugs on cell viability

**Figure 6:**
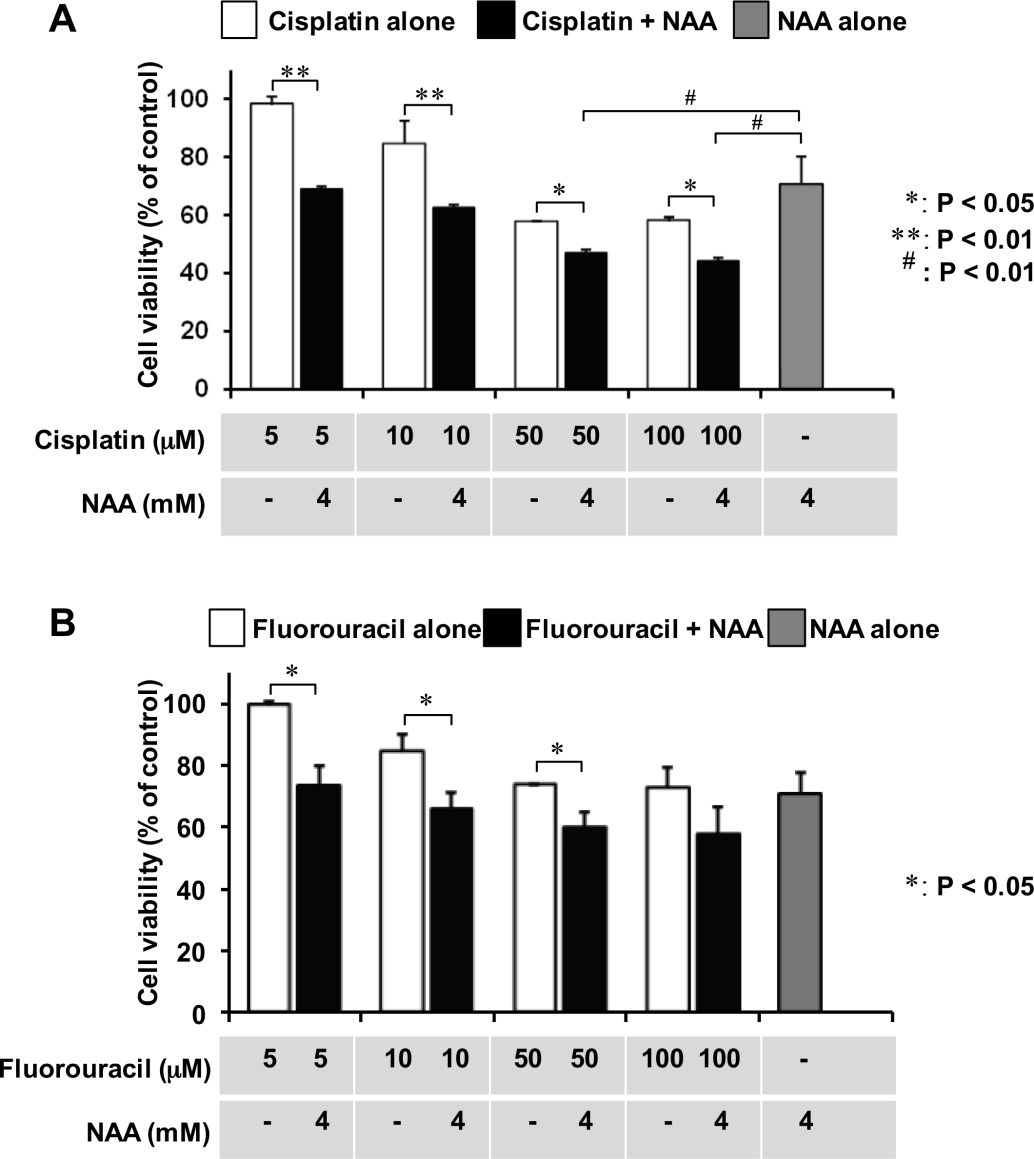
Effect of co-incubation of NAA and antineoplastic drugs on cell viability The cells were seeded at a density of 3 × 10^3^ cells/well into 96-well plates for 24 h. After preincubation with NAA (3 days), Cisplatin (**A**) or 5-Fluorouracil (**B**) was added to cells at the indicated concentrations. Cell viability was assessed by MTT method after 24 hr of Cisplatin and 48 hr of Fluorouracil addition. Data are expressed as mean ± SD of three independent experiments.

As differentiated cells exhibit a better responsiveness to drugs as compared with undifferentiated cells, we evaluated the sensitivity of SH-SY5Y cells to the antineoplastic agents Cisplatin and 5-fluorouracil, after treatment of the cell line with NAA. To this aim, we used a concentration of NAA and a time of treatment causing minor cytotoxic effects. SH-SY5Y cells were incubated in the presence of 4 mM NAA for 72 h and then treated with Cisplatin for 24 h and 5-fluorouracil for 48 h. As shown in Figure 6A and 6B, both Cisplatin and 5-fluorouracil caused in SH-SY5Y cells pre-treated with NAA a more significant decrease of the cell viability as compared with their effect on untreated cells.

## DISCUSSION

In this study, we sought to shed light on the function of NAA in the CNS. Although, it has long been recognized that NAA is the more abundant metabolite in brain, its role remains elusive. The specific ^1^H MRS signal of the acetyl (CH_3_) moiety makes NAA a well established biomarker of brain pathologies including tumours. Specifically in gliomas, astrocytomas, meningiomas as well as secondary metastasis, the decrease of the NAA-linked MRS signal in the tumor mass directly correlates with the severity of the neoplastic disease [32]. On this basis, NAA has been considered an oncosuppressor metabolite. In keeping with these premises, we investigated *in vitro* the impact of NAA treatment on the cell physiology of the neuroblastoma-derived SH-SY5Y subclonal cell line, a well established model for studying neuronal functions and differentiation.

As shown in Figure 1, 3-days treatment of SH-SY5Y cells with increasing physiological concentrations of NAA (i.e. in the mM range) resulted in progressive decrease of cell viability. It has to be mentioned that cultures of SH-SY5Y cells grow both as viable adherent and floating cells [33], moreover they comprise two phenotype subsets reminiscent of the neuroblast-like cells and epithelial-like cells present in the parental SK-N-SK cells [34]. The decreased viability of adherent cell following NAA treatment was shown to correlate with activation of the apoptotic program as highlighted by the positive reactivity to annexin V/PI as well as by the decrease of the anti-apoptotic factors Survivin and Bcl-XL. We did not further increase NAA-mediated cytotoxicity, but chose a concentration of NAA preserving 70% of cell viability for further analysis. MRS examination showed that, under the chosen experimental conditions, NAA was taken up by SH-SY5Y cells and metabolized (Figure 2). At sub-cytotoxic concentrations NAA caused a remarkable decrease of the cell growth. This observation was supported by a significant up-regulation of the major blockers of the cell cycle progression p53, p21^Cip1/Waf1^ and p27^Kip1^ (Figure 3).

Unexpectedly, already at 3 days treatment with 4 mM NAA, SH-SY5Y cells displayed morphological changes that became more evident after extending this treatment to 6 days (Figure 4). The neuroblastoma cells acquired an apparent neuronal morphology with long extensions like those induced by ATRA treatment, the most commonly used protocol for differentiation of SH-SY5Y cells to more mature neuron-like phenotype. In support of the morphological observation, the expression of the neurogenic markers TH and MAP2 was significantly increased.

To confirm that NAA-treatment induced genomic reprogramming in SH-SY5Y cells, a microarray expression analysis was carried out unveiling up-regulation and down-regulation in the expression of 260 and 115 genes respectively. The up-regulated genes included those coding for neuron markers and inhibitors of the cell cycle, whereas the down-regulated genes included hallmarks of undifferentiated cells (data not shown). The occurrence of stem cell-related markers has been found in different cancers, including neuroblastoma. The precise role of these stem cell-related genes in tumors is not completely clear, but Oct4 have been associated with a more immature and aggressive cell phenotype [35]. Consistent with this notion we found a significant decrease of Oct4 in NAA-treated SH, SY5Y cells, both the transcript and protein level as well as of CXCR4/CD184, another indifferentiation-state marker [36] (Figure 5).

Various lines of evidence suggest that stem-like cells are responsible for failure of long-term remission [37]. Thus, eradicating tumors may be difficult because conventional treatments target the bulk of tumor cells rather than these tumor-initiating stem cells which are chemoresistant. In the present study, we showed that co-treatment of SH-SY5Y cells with NAA and either Cisplatin or 5-fluorouracil resulted in a greater cytotoxic effect than those elicited by the two chemotherapeutic drugs alone. Most notably, combination of either of the chemotherapeutic drugs with NAA resulted in the same cytotoxic effect of that attained by the chemotherapeutic drugs alone but at a ten-fold lower dosage (cf. 10 μM drugs + 4 mM NAA vs 100 μM drugs alone in Figure 6). This result suggests that a dual therapy might be beneficial for improving the outcome of patients with high-risk neuroblastoma. In addition, our results support therapy using NAA alone in patients with low-stage disease.

Counterintuitively with the here-reported results, an increase of NAA has been, however, recently reported in non neural-derived solid tumors like non-small cell lung cancer [38] and ovarian cancer [39]. Moreover, NAA-mediated promotion of growth and inhibition of differentiation has been shown in glioma stem-like cells [40]. Such an apparent discrepancy might rely on a different tumor phenotype-dependent metabolic NAA-requirement. Consistently, a hitherto unknown function of NAA is emerging in lipid metabolism of non-nervous tissues like adipocytes [41]. In addition, the impact of NAA on neural-derived tumors might depend on their stemness status.

Although the mechanism of action has not been specifically addressed in this communication, it is tempting to speculate that this reported effect of NAA might be attributable to one or the other of the following: a) conversion of NAA in oxidizable products with a consequent diversion of the metabolism from glycolysis to oxidative phosphorylation with consequent activation of reactive oxygen species-mediated signaling; b) synthesis of the neurotransmitter NAAG; c) provision of acetyl-CoA which might promote epigenetic remodeling of chromatin as well as post-transcriptional modification of master transcription factors [42]. These possibilities are not mutually exclusive and can be easily tested and are amenable to further investigation in other tumour models.

In conclusion, given the urgency of finding alternative therapeutic strategies to treat aggressive neoplasia, such as neuroblastoma, our study provides a novel insight warranting further investigation in oncogenic studies of the nervous system.

## MATERIALS AND METHODS

### Preparation of drugs

Prior to use, NAA in the free acid form was diluted in fresh culture medium to the desired concentrations. Cisplatin and 5-FU were prepared fresh in water for injectable preparation and diluted in culture medium prior to use. The pH of the cell-incubating medium was checked for changes following addition of NAA resulting in minor variation (i.e. < 0.2 lowering of pH at the highest NAA concentration used in this study).

### Cell cultures and toxicity studies

SH-SY5Y cells were cultured at 37°C, with 5% CO2 in DMEM/F12 medium (Sigma Aldrich, St.Louis, MO) supplemented with 10% fetal bovine serum (FBS; Sigma Aldrich, St.Louis, MO), 100 IU/ml penicillin (Sigma Aldrich, St.Louis, MO), 100 μg/ml streptomycin (Sigma Aldrich, St.Louis, MO) and glutamine (2 mM) (Sigma Aldrich, St.Louis, MO). Cells (2–2.5 × 10^6^ per 10 mm plates, 3 × 10^3^ per a 96-well microtiter plate) were incubated with growth medium for 24 h. One day after seeding, NAA (4 × 10^−3^ M) was added to the cells and 72 h after treatment, cells susceptibility was evaluated. Following 72 h of NAA treatment, cells were exposed to different concentrations of Cisplatin (5 × 10^−6^ to 1 × 10^−4^ M) and 5-FU (5 × 10^−6^ to 1 × 10^−4^ M) and cell viability was assessed 24 h and 48 h later, respectively. Control cells were incubated with fresh medium or NAA alone.

### Cell viability assay

Cells were seeded in a 96-well culture plate and, after 72 h of 4 mM NAA treatment, cell viability was measured by MTS assay as described in [43]. Three independent measurements were performed in triplicate for each assay.

### Apoptosis assay

After 72 h 4 mM NAA treatment, SH-SY5Y cells were stained with Annexin-V-FITC and PI (BD Biosciences). Apoptotic and necrotic cells were detected by flow citometry (FACScantoII, Becton Dickinson). Three independent experiments were carried out. A total of 10^4^ events for each sample were acquired.

### Nuclear magnetic resonance spectroscopy of cultured cells

The examinations were performed on a 3T MRI unit (Achieva, Philips Medical Systems, Best, The Netherlands) equipped with gradients of amplitude and maximum slew rate respectively of 80 mT/m and 200 mT/m/ms, using an eight-channel receiver head coil. Automatic dynamic high-order shimming and chemical-shift selective water suppression using Gaussian pulses were used. Voxel sizes for 2D PRESS studies were typically 1 × 1 × 1 cm. The spectra were automatically analyzed for the relative signal intensities (areas under the fitted peaks in the time domain) of the following metabolites the creatine–phosphocreatine complex (Cr) and N-acetyl aspartate (NAA). The ratio NAA/Cr was calculated at TE = 288 ms. Postprocessing steps, including frequency shift, baseline correction, phase correction, and peak fitting/analysis, were performed first automatically and then manually if necessary using the software package provided by the manufacturer (SpectroView, Philips Medical Systems, Best, the Netherlands). All spectral analyses were performed in a window from 0.50 to 4.30 ppm (using the standard method of assigning a shift value of 4.7 ppm to the measured unsuppressed water peak). The metabolites were quantified from a single representative voxel. We considered that peaks at 1.3 and 0.9 ppm primarily consisted of lipids and the peak at 1.4 ppm consisted of signal from lactate.

### RNA extraction, reverse transcription and real-time polymerase chain reaction analysis

Total cellular RNA was processed and 1 μg of total RNA was retro-transcribed and the cDNA was used to perform a real-time PCR reaction as described in [44] using POU5F1 TH, MAP2 and GAPDH primers as as reported in [45] and in the Table 1 of Supplementary data. For TH and MAP2 genes, the following primers protocols were used for the PCR reactions, initialization at 95°C for 3 min followed by 40 cycles of 95°C for 10 s, 57°C (for TH) and 56°C (for MAP2) for 10 s, 72°C for 10 s. The melting program was 95°C for 5 s, 65°C for 1 min and 97°C for 10 s. The rate of temperature increase was 1°C/s and fluorescence was continuously acquired. The relative amounts of target genes were normalized to GAPDH expression by Light Cycler^®^ 480 Software version 1.5 (Roche Diagnostics) using the 2^−ΔΔCt^ method.

### Western blotting

Aliquots containing 30 μg of proteins from each lysate cells were subjected to sodium dodecyl sulfate polyacrylamide gel electrophoresis on a 4–20% gradient gel under reducing conditions and then electrotransferred onto a polyvinylidene difluoride membrane using the Trans Blot Turbo Transfer System (Bio-Rad Laboratories, Hercules, CA, USA). Membranes were probed with primary antibodies Oct4 (1:500; Abcam Cambridge, UK), Bcl-xL (1:1000; Cell Signaling Technology, Beverly, MA, USA), Survivin (1:1000; Cell Signaling Technology, Beverly, MA, USA), p53 (1 1000; Cell Signaling Technology, Beverly, MA, USA), p21^Cip1/Waf1^ (1:1000; Millipore, Billerica, MA, USA), p27^Kip1^ (1:500; Abcam Cambridge, UK) and then incubated with secondary antibody (horseradish peroxidise-conjugated goat anti-mouse or anti-rabbit 1:2500; Cell Signaling, Beverly, MA, USA). The same membranes were then stripped with Actin (mouse anti-Actin antibody 1:5000; Sigma Aldrich, St.Louis, MO). Immune complexes were detected by the ECL chemiluminescence system (Bio-Rad Laboratories, Hercules, CA, USA), as recommended by the manufacturer.

### Co-expression cytofluorimetric analysis of cell surface marker and intracellular antigens

Cell suspensions (0.8–1 × 10^6^ cells/ml per condition) were stained with CD184-allophycocyaninm (APC,) directly conjugated antibody (1:50; eBioscience) in the dark at room temperature for 30 min, followed by fixation, permeabilization and subsequent indirect staining for intracellular antigens, as described in [46]. Primary antibodies MAP2 (1 : 50; Cell Signaling Technology) and TH (1 : 100; Cell Signaling Technology) were incubated in the dark with 10% fetal bovine serum and 1% bovine serum albumine (BSA) in PBS and then incubated with secondary antibody Alexa Fluor 488 (anti-rabbit, 1:1000; Life Technologies). For washing steps, the supernatant was removed by decanting, allowing a minor volume of liquid (ca. 100 μl) to remain. After washing, a total of 10^4^ events for each sample was acquired and analysed by FACS Calibur flow cytometer with CellQuest software (BDB).

### Statistical analysis

Experimental data are reported as mean ± standard deviation or mean ± standard error of the mean. Data were compared by an unpaired Student's-*t*-test. Differences were considered statistically significant when the *P* value was less than 0.05. All analyzes were performed using Graph Pad Prism (Graph Pad Software, San Diego, CA, USA).

## SUPPLEMENTARY MATERIALS TABLE


