# Supplementary material for: N-acetylaspartate (NAA) induces neuronal differentiation of SH-SY5Y neuroblastoma cell line and sensitizes it to chemotherapeutic agents
Source: Oncotarget. 2016 Mar 29;7(18):26235–46. doi: 10.18632/oncotarget.8454 (PMC5041977; doi:10.18632/oncotarget.8454)
Supplement: Supplementary file 1 [file oncotarget-07-26235-s001.pdf]

N-acetylaspartate (NAA) induces neuronal differentiation of SH-SY5Y neuroblastoma cell line and sensitizes it to chemotherapeutic agents

Supplementary Materials

Supplementary Table S1: Features of the primers used for real-time polymerase chain reaction

Transcript	T_{ann} (◦C)	Length (bp)	NCBI Ref Seq or sequence
POU5F1	60	77	NM_203289
TH **[27]**	57	90	Fw:GCCCTACCAAGACCAACGTA<br>Rv:CGTGAGGCATAGCTCCTGA
MAP2 **[27]**	56	209	Fw:CATGGGTCACAGGGCACCTATTC<br>Rv:GGTGGAGAAGGAGGCAGATTGCTG
GAPDH	60	135	Fw:AGGCTGAGAAACGGGAAGC<br>Rw:CCATGGTGGTGAAGACGC

NCBI Ref Seq: National Center for Biotechnology Information Reference Sequence (http://www.ncbi.nlm.nih.gov/RefSeq/) identifier.
